# Training the healthcare workforce: the global experience with telementorship for hepatitis B and hepatitis C

**DOI:** 10.1186/s12913-023-09849-y

**Published:** 2023-08-02

**Authors:** Maria A. Corcorran, Karla Thornton, Bruce Struminger, Philippa Easterbrook, John D. Scott

**Affiliations:** 1 Division of Allergy and Infectious Diseases, Department of Medicine, University of Washington, Seattle, WA USA; 2 Division of Allergy and Infectious Diseases, Department of Medicine, University of Washington School of Medicine, Harborview Medical Center, 325 9th Ave, Box 359782, Seattle, WA 98104 USA; 3 Division of Infectious Diseases, Department of Internal Medicine, University of New Mexico, Albuquerque, NM USA; 4 Project ECHO, University of New Mexico, Albuquerque, NM USA; 5 Department of Global HIV, Hepatitis and STI Programmes, World Health Organization, Geneva, Switzerland

**Keywords:** Hepatitis B, Hepatitis C, Telehealth, Telementorship, Project ECHO, Hepatitis elimination, Healthcare worker training

## Abstract

**Background:**

Telementorship has emerged as an innovative strategy to decentralise medical knowledge and increase healthcare capacity across a wide range of disease processes. We report the global experience with telementorship to support healthcare workers delivering hepatitis B virus (HBV) and hepatitis C virus (HCV) care and treatment.

**Methods:**

In early 2020, we conducted a survey of HBV and HCV telementorship programmes, followed by an in-depth interview with programme leads. Programmes were eligible to participate if they were located outside of the United States (U.S.), focused on support to healthcare workers in management of HBV and/or HCV, and were affiliated with or maintained adherence to the Project ECHO model, a telementorship programme pioneered at the University of New Mexico. One programme in the U.S., focused on HCV treatment in the Native American community, was purposively sampled and invited to participate. Surveys were administered online, and all qualitative interviews were performed remotely. Descriptive statistics were calculated for survey responses, and qualitative interviews were assessed for major themes.

**Results:**

Eleven of 18 eligible programmes completed the survey and follow up interview. Sixty-four percent of programmes were located at regional academic medical centers. The majority of programmes (64%) were led by hepatologists. Most programmes (82%) addressed both HBV and HCV, and the remainder focused on HCV only. The median number of participating clinical spoke sites per programme was 22, and most spoke site participants were primary care providers. Most ECHO sessions were held monthly (36%) or bimonthly (27%), with sessions ranging from 45 min to 2 h in length. Programme leaders identified collective learning, empowerment and collaboration to be key strengths of their telementorship programme, while insufficient funding and a lack of protected time for telementorship leaders and participants were identified as major barriers to success.

**Conclusion:**

The Project ECHO model for telementorship can be successfully implemented across high and low-and-middle-income countries to improve provider knowledge and experience in management of viral hepatitis. There is a tremendous opportunity to further expand upon the existing experience with telementorship to support non-specialist healthcare workers and promote elimination of viral hepatitis.

**Supplementary Information:**

The online version contains supplementary material available at 10.1186/s12913-023-09849-y.

## Background

Globally, an estimated 296 million people are chronically infected with hepatitis B virus (HBV) and 58 million with hepatitis C virus (HCV), the majority of whom reside in low-and-middle-income countries (LMICs) [1]. Despite the availability of effective suppressive therapy for HBV and curative therapy for HCV, in 2019 the World Health Organization (WHO) estimated that only 2% of persons who met criteria for HBV treatment were on antiviral therapy, and only 13% of those diagnosed with chronic HCV had been treated [1]. Disparities in the cascade of care for viral hepatitis are particularly pronounced in rural and underserved areas, where a lack of experienced healthcare providers, among many other socioeconomic factors, limits the access to care and treatment [2, 3]. Task-shifting to non-specialists and care decentralisation were widely adopted as strategies to increase workforce capacity in the global response to HIV and represent opportunities for the expanded delivery of viral hepatitis care, particularly in lower resourced settings [4–8]. Nevertheless, prior studies indicate that knowledge of viral hepatitis prevention, screening and management remains low among frontline healthcare workers [9–16], and additional efforts are needed to increase workforce training and further facilitate decentralisation of care.

Telementorship has emerged as an effective strategy to increase healthcare workforce capacity across a variety of fields and subspecialties, with Project ECHO (Extension for Community Health Outcomes), being the most widely studied model for telementorship, particularly in the treatment of viral hepatitis [17, 18]. Project ECHO was developed by the University of New Mexico (UNM) in 2003 as a platform to improve HCV care delivery and access to care for minority and underserved populations [17, 19]. The model utilizes videoconferencing technology to connect specialist teams, often at regional or academic centers (known as hub sites), to primary care providers, nurses, pharmacists, community health workers and other non-specialists in rural and/or underserved locations (known as spoke sites) [20]. Virtual ECHO sessions are typically held weekly to monthly. During these sessions, specialty teams at the hub site review and discuss patient cases presented by primary providers at the spoke sites using de-identified patient information [21]. In addition, during these sessions, specialty providers may also present a short didactic lecture on a topic pertinent to the group’s clinical practice. Through this knowledge sharing network and community of practice, primary care providers gradually develop increasing levels of knowledge, comfort, independence and self-efficacy in the management of complex disease processes and can serve as champions within their own communities [20, 21].

Although Project ECHO was initially developed by UNM to improve the care of people and communities living with HCV in rural New Mexico, over the years the model has been adapted to improve access to care across various medical specialties and geographic locations [2, 3, 20, 22–34]. In particular, experience with Project ECHO’s telementorship model has grown substantially over the past three years, as the platform has been successfully leveraged to address the evolving COVID-19 pandemic [35–37] and used to adapt clinical services for other disease processes as a result of social distancing measures at healthcare facilities [38]. As of February 2022, 58 countries had at least one ECHO partner hub across multiple specialties [39]. Viral hepatitis ECHO programmes are well represented globally, with 53 partner programmes in 13 countries including: Argentina, Australia, Brazil, Canada, Chile, Egypt, Georgia, India, Pakistan, South Africa, the United States (U.S.), Uruguay, and Zambia [40].

In response to the need for increased decentralisation of viral hepatitis care and provider knowledge, we convened a global viral hepatitis workforce training steering group to systematically identify, collate and critically review available resources and curricula for viral hepatitis workforce training, including models for provider mentorship in LMICs, such as Project ECHO. In doing so, we established a framework for viral hepatitis training, which emphasized the distinct phases of healthcare worker training, including: 1. Initial training and 2. Ongoing mentorship and support.

We considered initial training to encompass online trainings, in-person and virtual workshops, onsite clinical training, and other supportive educational materials that provide healthcare workers with a comprehensive, often one-time, introduction to the management of HBV and HCV. In this article we focus on the second phase of provider training – ongoing mentorship and support – under which we identified telementorship, mobile messaging groups, e-consults and online discussion forums. This article specifically discusses the rapidly growing innovation of telementorship, focusing on Project ECHO. Through surveys and qualitative interviews with viral hepatitis telementorship programmes across the world, we sought to understand the global experience with telementorship for the management of HBV and HCV and describe key operational and programmatic features, including successes and challenges, in an effort to inform the development and expansion of viral hepatitis telementorship programmes globally.

## Methods

### Study design and data source

This was an exploratory mixed-methods study, in which we conducted a cross-sectional survey of HBV and HCV telementorship programmes, followed by an approximately 30-min qualitative interview with survey respondents. Telementorship programmes were eligible for inclusion if they were located outside of the U.S., focused on training healthcare workers to provide care for HBV, HCV, or both, and were formally affiliated with Project ECHO at UNM or maintained adherence to the ECHO model approach despite a lack of formal affiliation. Due to the high number of viral hepatitis ECHO programmes within the U.S., U.S.-based programmes were excluded so as to not bias the sample towards high-income countries. The one exception to this was a U.S.-based programme focusing on the care of the Cherokee Native American population. This programme was purposively sampled to gain a better understanding of how Project ECHO can be used to increase healthcare capacity within native and indigenous populations, given disproportionately high rates of viral hepatitis and structural barriers to care in these communities [41–45].

UNM’s Project ECHO administrative office provided a complete listing of partner programmes outside of the U.S., which was used to identify eligible programmes. The list was initially collated in June of 2019 and further updated in January of 2020. Additional programmes were identified through a global working group on viral hepatitis training. In total, we identified 15 viral hepatitis Project ECHO programmes outside of the United States through information provided by the Project ECHO administrative offices at UNM. Through our global steering group network, we identified two additional telementorship programmes in India that had not been identified by Project ECHO but maintained adherence to the Project ECHO model for telementorship. We additionally included one programme in the U.S., providing telementorship services to the Cherokee Native American Nation, to enable inclusion of information on the experience of telementorship in supporting viral hepatitis care for indigenous populations, as outlined above.

### Data collection and measures

An online survey was developed by a core committee of investigators from the World Health Organization (WHO), Project ECHO – UNM, and the University of Washington (UW). The survey included questions on demographic information, the clinical setting in which the telementorship programme was located, the state of the national viral hepatitis response in the country where the telementorship programme was located, telementorship programme activities, operational aspects of the telementorship programme, and reflections on the telementorship programme thus far (Additional file 1: Appendix A). After survey questions were agreed upon by the core investigational team, a survey instrument was built using the Research Electronic Data Capture (REDCap) software programme (version 9.4.2 © 2019 Vanderbilt University) [46, 47]. An online public survey link was sent via email to all eligible telementorship programmes, and responses were collected automatically in REDCap from January 2020 through March 2020. Any respondent affiliated with the ECHO programme was eligible to participate. Participation was voluntary, and no incentive was provided.

A 12-item structured interview guide, focusing on telementorship programme evolution, operations, challenges, successes and future directions, was similarly develop by the core committee of investigators from the WHO, Project ECHO – UNM, and the UW (Additional file 2: Appendix B). Telementorship programmes who completed the online REDCap survey, were then invited to participate in an interview. Interviews were conducted remotely utilizing the teleconferencing platform Zoom between January and March 2020. All interviews were conducted by one study investigator (M.C.) who was knowledgeable about telementorship and had experience participating in Project ECHO. Interviews lasted approximately 30 min but varied in length. Participation in interviews was voluntary, and no incentive was provided. Structured notes of each interview were taken and entered into a templated data collection instrument within REDCap by one study investigator (M.C.) during and immediately following each interview. During the interview process, programmes with incomplete survey data were asked to provide responses for any missing data points, and therefore accommodations for missing data were not needed.

### Statistical analysis

Basic descriptive and summary statistics were performed, when appropriate, for survey questions. All data was stored in REDCap (version 9.4.2 © 2019 Vanderbilt University), and statistical analyses were performed using R Studio (© R Foundation for Statistical Computing, 2016). Structured notes for each individual interview were taken, as described above, and then downloaded in PDF format from REDCap. Interview responses were reviewed and manually coded by one member of the study team (M.C.). Major themes were identified apriori based on the interview guide, with emergent themes identified thoughout the analysis. Interview responses were initially reviewed with a focus on major themes (e.g., programmatic strengths, positive features, major challenges, barriers to success, and future directions) and then iteratively reviewed for emergent themes. Results were then summarized by M.C. and triangulated with responses to open ended survey questions.

### Ethics

ECHO programmes were sent an email that provided information on the general content, logistics and aims of the survey and interview. Programmes that completed the survey provided verbal consent to participate in the follow-up interview. Because the content of the survey and interviews focused on ECHO programs and not individuals, guidance from the University of Washington Human Subjects Division allowed for a determination that this research did not involve human subjects, and therefore formal IRB review was not required.

## Results

**Fig. 1:**
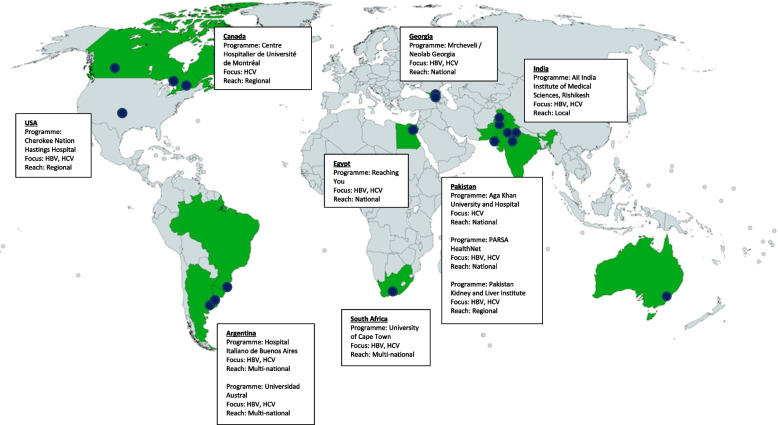
Map of eligible telementorship programs. Telementorship programs eligible for this study are indicated by individual blue dots. Information on programs that participated in surveys and interviews is provided in the text boxes, organized by country

In total, 18 telementorship programmes, 6 in the WHO Eastern Mediterranean Region (Pakistan [*n* = 3]; Republic of Georgia [*n* = 2]; Egypt [*n* = 1]), 3 in the South-East Asia Region (India [*n* = 3]), 1 in the Western Pacific Region (Australia [*n* = 1]), 1 in the Africa Region (South Africa [*n* = 1]), and 7 in the Region of the Americas (Brazil [*n* = 1]; Argentina [*n* = 2]; Canada [*n* = 3]; U.S. [*n* = 1]), (Fig. 1), were sent a weblink to the online telementorship programme survey. Eleven programmes completed the survey and were included in our analysis. All 11 programmes participated in the subsequent interview to discuss qualitative aspects of their telementorship programme with a study investigator. Programs that did not respond to the survey invitation were geographically dispersed and included 1 program in Georgia, 2 programs in India, 1 program in Australia, 1 program in Brazil, and 2 programs in Canada.

### Description of telementorship programme clinical setting

Key programmatic and operational features of each telementorship programme are summarized in Table 1. The majority of programmes who completed the survey (*n* = 7, 64%) were located at regional academic medical centers, with the remaining 4 programmes located within a private clinical practice, a non-governmental organization, a tribal healthcare system, and an independent educational initiative. Among the ECHO programmes surveyed, there was a median of 3 (interquartile range [IQR] 2.5 – 7.0) specialist physicians, 4 (IQR 0.5 – 6.5) general physicians, and 3 (IQR 0 – 7) nurses in their hepatitis clinical practices. These clinical practices received referrals from a median of 15 (IQR 9.0 – 21.5) hospitals or primary care clinics.

**Table 1:** Viral hepatitis telementorship programmes

ECHO Hub	Hub Location	Type of institution: hub		Year of Initiation	Attended ECHO Immersion (Y/N)	Current number of spoke sites	Predominant location of spoke cites (urban, rural)	Frequency of ECHO Clinics	Leader of ECHO clinics at hub	Provider type at spoke sites	Cover HBV and/or HCV	Education credits or certificate?	Provide workshops, online trainings, mobile messaging groups, other?	Video conferencing platform
**Southeast Asian Region**														
AII India Institute of Medical Sciences	Rishikesh, India	Academic medical center		2019	Yes	26	50% rural	Monthly	Team of epidemiologists, gastroenterologists and microbiologists	General practitioners Nurses Pharmacists Community health workers Medical and other health professional students Lab technicians Data entry operators	HBV HCV	Certificate of competency	WhatsApp group Workshops Online trainings	Zoom
**Eastern Mediterranean Region**														
Aga Khan University & Hospital	Karachi, Pakistan	Academic medical center		2017	Yes	10	100% urban or semi-urban	Twice monthly	Hepatologist	General practitioners Nurses Pharmacists Clinic admin staff	HCV	Certificate of competency	WhatsApp Workshops	Zoom
Pakistan Kidney and Liver Institute	Lahore, Pakistan	Tertiary referral hospital		2018	Yes	24	90% rural	Weekly	Hepatologist	General practitioners	HBV HCV	No	WhatsApp group Workshops	Zoom
PARSA HealthNet	Gujranwala, Pakistan	Not for profit outpatient liver clinic		2017	Yes	50	60% urban or semi-urban	Weekly	Hepatologist	General practitioners Liver specialists Nurses Medical/health professional students	HBV HCV	CME Certificate of completion	WhatsApp group	Zoom
Mrcheveli – Georgia ID/AIDS_HEPA Center	Tbilisi, Georgia	Outpatient subspecialty clinic		2016	Yes	8	100% urban or semi-urban	Every other month	Hepatologist	General practitioners Liver specialists ID specialist	HBV HCV	No	Phone consultation	Zoom
Reaching You	Cairo, Egypt	Independent web-based initiative		2015	Yes	3	100% rural	On demand	Public health professional	General practitioners	HBV HCV	No	Send out reports and educational materials to spokes	Zoom
**Region of the Americas**														
Universidad Austral	Pilar, Argentina	Academic medical center		2015	No	22	75% urban or semi-urban	Monthly	Hepatologist	General practitioners Liver specialists ID specialists	HBV HCV	No	WhatsApp Workshops	Zoom
Hospital Italiano de Buenos Aires	Buenos Aires, Argentina	Academic medical center		2015	Yes	30	85% urban or semi-urban	Monthly	Hepatologist	General practitioners Liver specialists ID specialists	HBV HCV	No	No	Zoom
Université de Montréal	Montreal, Canada	Academic medical center		2017	Yes	51	75% urban or semi-urban	Twice monthly	Alternates between hepatologist, ID specialist, nurse and general practitioner	General practitioners ID specialists Nurses Community health workers Medical/health professional students	HCV	CME	No	Zoom
Cherokee Nation Health Services	Tahlequah, Oklahoma, USA	Tribal-operated hospital and health system		2014	Yes	12	100% rural	Twice monthly	ID specialist	General practitioners Nurses Pharmacists Social workers	HBV HCV	No	Workshops	Skype
**African Region**														
University of Cape Town	Cape Town, South Africa	Academic medical center		2019	Yes	11	100% urban or semi-urban	Monthly	Hepatologist	General practitioners Liver specialists	HBV, HCV	No	Written reports	Zoom

### Current status of national hepatitis response

Of the telementorship programmes surveyed, all but one reported some form of national plan for viral hepatitis elimination; although many programmes reported there were large programmatic barriers to the success of their national plan, including a lack of funding and coordination. Fifty-five percent (*n* = 6) of programmes reported viral hepatitis treatment in their country was being provided through both the public and private sector, with the remaining 45% reporting viral hepatitis treatment was predominantly provided through the public sector (Table 2). All telementorship programmes reported that all liver disease specialists in their country were able to prescribe treatment for HBV and HCV, and 82% and 73% of countries reported that infectious disease specialists could also prescribe HBV and HCV treatment, respectively. A similar proportion of programmes reported that general practitioners could prescribe treatment for HBV (73%) and HCV (82%). No programmes reported that viral hepatitis treatment could be directly prescribed by pharmacists or nurses.

**Table 2:** Status of national hepatitis elimination plan

ECHO Programme	Hub Location	National plan for viral hepatitis elimination (Y/N)		Primary provider of viral hepatitis care (private vs public sector)		Who can prescribe antivirals for HBV?		Who can prescribe antivirals for HCV?	Antivirals in use for HBV		Antivirals in use for HCV
**Southeast Asian Region**											
AII India Institute of Medical Sciences	Rishikesh, India	Yes		Public sector		Liver specialists ID specialists General practitioners		Liver specialists ID specialists General practitioners	TDF TAF ETV		SOF/VEL LED/SOF SOF + DAC INF + RBV
**Eastern Mediterranean Region**											
Aga Khan University & Hospital	Karachi, Pakistan	Yes		Public and private		Liver specialists ID specialists General practitioners		Liver specialists ID specialists General practitioners	TDF TAF ETV		SOF/VEL SOF + DAC
Pakistan Kidney and Liver Institute	Lahore, Pakistan	Yes		Public and private		Liver specialists ID specialists General practitioners		Liver specialists ID specialists General practitioners	TDF ETV		SOF/VEL SOF + DAC INF + RBV
PARSA HealthNet	Gujranwala, Pakistan	Yes		Public and private		Liver specialists General practitioners		Liver specialists General practitioners	TDF TAF ETV		SOF/VEL SOF + DAC
Mrcheveli – Georgia ID/AIDS_HEPA Center	Tbilisi, Georgia	Yes		Public and private		Liver specialists ID specialists		Liver specialists ID specialists General practitioners	TDF		SOF/VEL LED/SOF
Reaching You	Cairo, Egypt	Yes		Public sector		Liver specialists		Liver specialists	TDF ETV		SOF + DAC
**Region of the Americas**											
Universidad Austral	Pilar, Argentina	Yes		Public and private		Liver specialists ID specialists General practitioners		Liver specialists ID specialists General practitioners	TDF TAF ETV		G/P SOF/VEL LED/SOF SOF/VEL/VOX SOF + DAC EBR/GZR
Hospital Italiano de Buenos Aires	Buenos Aires, Argentina	Yes		Public sector		Liver specialists ID specialists General practitioners		Liver specialists ID specialists General practitioners	TDF TAF ETV		G/P SOF/VEL LED/SOF SOF/VEL/VOX SOF + DAC EBR/GZR
Université de Montréal	Montreal, Canada	No		Public sector		Liver specialists ID specialists General practitioners Specialized nurse practitioners		Liver specialists ID specialists General practitioners Specialized nurse practitioners	TDF TAF ETV		G/P SOF/VEL LED/SOF SOF/VEL/VOX SOF + DAC EBR/GZR INF + RBV
Cherokee Nation Health Services	Tahlequah, Oklahoma, USA	Yes		Public and private		Liver specialists ID specialists General practitioners		Liver specialists ID specialists General practitioners	TDF TAF ETV		G/P SOF/VEL LED/SOF SOF/VEL/VOX EBR/GZR
**African Region**											
University of Cape Town	Cape Town, South Africa	Yes		Public sector		Liver specialists ID specialists General practitioners		Liver specialists	TDF TAF ETV 3TC		SOF/VEL LED/SOF SOF/VEL/VOX SOF + DAC

The most common direct acting antivirals (DAAs) in use in country for the treatment of HCV were sofosbuvir/velpatasvir (91%) and sofosbuvir plus daclatasvir (82%). Of the programmes surveyed, 36% reported availability of glecaprevir/pibrentasvir, 64% ledipasvir/sofosbuvir, 45% sofosbuvir/velpatasvir/voxilaprevir, and 36% elbasvir/grazoprevir. All programmes reported the use of tenofovir disoproxil fumarate (TDF) for the treatment of chronic HBV in country, with 73% also reporting the use of tenofovir alafenamide (TAF), and 91% the use of entecavir. Laboratory-based HCV RNA and HBV DNA testing was in use in 91% and 100% of countries, respectively.

### Description of ECHO programme and activities

Four (36%) programmes reported they had national coverage (e.g. spoke sites located across the country), 3 (27%) multi-national coverage (e.g. spoke sites located in other countries), 3 (27%) a regional scope, and 1 (9%) a local area scope. Most programmes addressed both HBV and HCV (*n* = 9, 82%), and the remainder addressed HCV only. Surveyed programmes had a median of 22 current spoke sites (IQR 10.5 – 28.0); although most started with far fewer (median 10, IQR 3 – 16). Seven programmes (64%) reported their spokes sites were predominantly located in urban or semi-urban centers, and 3 (27%) had spokes sites predominantly in rural areas. One programme (9%) reported an equal split between urban or semi-urban and rural spoke sites. There was a diversity in the type of spoke sites participating in Project ECHO, with most hubs training primary care or general medicine clinics (*n* = 10), specialty clinics (*n* = 5), and government hospitals (*n* = 5). Only one programme reported training harm reduction and/or syringe service programmes, addiction medicine clinics, and jail or prison health clinics.

### Operational aspects

There was some variability in the frequency of telementorship clinics including: every other month (*n* = 1, 9%), monthly (*n* = 4, 36%), twice a month (*n* = 3, 27%), and weekly (*n* = 2, 18%). One programme reported meeting on an as needed basis, coordinating with hub experts and spoke sites. All but one programme utilized the Zoom videoconferencing platform to run their telementoring clinics. Surveyed programmes reported that their educational session length ranged from 45 min to 2 h, with most programmes (*n* = 7, 64%) reporting hour-long sessions. The majority of telementorship sessions were led by hepatologists (*n* = 7, 64%); although other panel members at the hub sites included infectious disease physicians (27% of programmes), general physicians (9% of programmes), nurses (27% of programmes), pharmacists (18% of programmes), social workers (9% of programmes), and community health workers (9% of programmes). The time staff members dedicated to ECHO activities varied across sites. Several sites reported no dedicated salary support or administrative support for ECHO activities, while others had part-time administrators and protected time for supporting ECHO learning activities. ECHO leaders reported spending anywhere between a few hours per session to 20% of their total work time on ECHO-related activities.

**Fig. 2:**
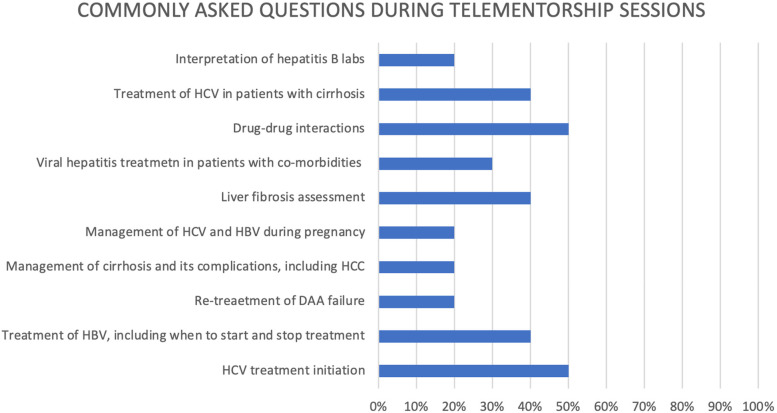
Commonly asked questions during telementorship sessions

Among surveyed telehealth programmes, a median of 18 (IQR 11—20) spoke site participants attended each telehealth clinic. Programmes reported that attendees at spoke sites routinely included: general practitioners / primary care doctors (100% of programmes), liver specialists (45% of programmes), specialists in infectious diseases (36% of programmes), nurses (45% of programmes), pharmacists (27% of programmes), social workers (9% of programmes), community health workers (18% of programmes), and medical or other health professional students (36% of programmes). One programme reported that lab technicians and data entry operators attended their telementorship clinics, while another programme reported that clinic administrative staff attended. Among the programmes surveyed, the five most common clinical questions asked during ECHO learning sessions were related to: 1. Drug-drug interactions; 2. HCV treatment initiation; 3. Treatment of HCV in patients with cirrhosis; 4. Treatment of HBV, including when to start and stop therapy; 5. Assessing liver fibrosis (Fig. 2).

Five telementorship programmes (45%) used a complementary mobile messaging group (predominantly via WhatsApp) to respond to provider questions between ECHO sessions, and 4 programmes provided in-person workshop trainings, with one additional programme offering both in-person workshops and online trainings to their ECHO participants. Two programmes offered ECHO participants continuing medical education (CME) credits for participating in the telementorship programme, and 3 programmes offered a certificate of competency.

### Programme initiation and funding

Of the 11 telementorship programmes surveyed, all were started between 2014 and 2019. In qualitative interviews, most programmes reported that they had established an ECHO programme in response to: 1. A lack of viral hepatitis provider education in the local, regional or national medical community; 2. A high baseline prevalence of HBV and/or HCV in their community; and 3. The need for increased HBV and HCV testing and treatment capacity, particularly following the introduction of DAAs. In addition, several programmes, including the Georgia Ministry of Health HCV ECHO Programme and the Uttarakhand Viral Hepatitis ECHO Programme in India, were developed as an integral part of the government’s national or regional plan for viral hepatitis elimination.

Prior to initiation, the majority of programme leads (*n* = 10, 91%), attended a 3-day ECHO Immersion course at the University of New Mexico or at another global ECHO Superhub [48]. Sources of funding for the telementorship programmes surveyed mainly consisted of corporate pharmaceutical support (*n* = 6, 56%). One programme received money from the local government, one from their parent university, and one from a not-for-profit organization. Three programmes reported no external funding support.

### Programme evolution

Since their inception, all ECHO hubs reported an increase in the number of spoke sites affiliated with their programme, with the addition of a median of 9 spoke sites (IQR 7.0 – 14.5) per hub. While many programmes started out providing telementorship specific to HCV, almost all had expanded to include mentorship support for HBV at the time of survey completion. Additionally, several programmes, such as Austral University in Argentina, reported starting separate ECHO programmes for advanced liver disease, including management of cirrhosis, liver transplantation and hepatocellular carcinoma. Expanding knowledge of viral hepatitis and liver disease across the spoke sites has allowed programmes like the Hospital Italiano de Buenos Aires Viral Hepatitis ECHO programme to cover more cases per ECHO session, and several programmes noted, that with increased knowledge came increased participation among providers at the spoke sites.

### Data collection

Ninety-one percent of programmes surveyed reported maintaining some form of data on their programme operations or outcomes. The types of data collected were broad and fell into the following general categories: spoke site demographics; ECHO attendance; clinical questions asked during ECHO sessions; patient level demographics; recommendations provided to spoke sites; number of patients screened, treated and cured through the ECHO programme; and provider knowledge assessments. Several institutions have published data from their ECHO programmes over the past several years, including the Austral University Viral Hepatitis ECHO Programme [2], the Viral Hepatitis ECHO Programme at Hospital Italiano de Buenos Aires [29], and the Cherokee Nation Health Services Hospital and Clinics [49, 50]. Similarly, Georgia’s HCV elimination programme, which has utilized Project ECHO to help train HCV treatment programme providers, has published data on treatment outcomes among patients treated through their national programme [51].

### Reflections on successes, challenges, and future directions

In qualitative interviews, programmes identified several positive features and attributes of their telementorship programmes. The key strengths highlighted by programme leaders related to learning, empowerment and collaboration. Nearly all programme leaders mentioned that the Project ECHO model was highly effective in promoting learning at the spoke sites, and many programme leaders were surprised by how much the hubs learned from the spokes. Several hub leaders noted that with increasing knowledge, the spoke sites and healthcare workers became progressively empowered to manage HBV and/or HCV infection independently and to take on higher volumes of patients. Additionally, hub leaders identified collegiality and collaboration as a positive feature of their programme and the Project ECHO model. Through the hub and spoke collaboration, many hub leaders felt they were able to build trust and rapport with the spokes. This in turn created a collegial and collaborative environment, promoting group learning and ultimately improvements in patient care.

In addition to strengths and positive features of their telementorship programme, ECHO hub leaders also identified various challenges and barriers to success. One of the biggest challenges identified across ECHO programmes was the lack of protected time for spoke site providers to participate in the programme. Several hub leaders remarked that it was often challenging for spoke site participants to attend ECHO sessions and prepare and present cases, as they did not have protected time within their schedule to do so. A lack of protected time was a similar challenge for faculty on the hub side, where many programmes reported difficulties with securing adequate funding to support their telementorship efforts, and several hub leaders effectively volunteered their time to support programme operations. Several programmes also reported shyness among the spoke providers to be a barrier to participation; a lack of government support as a barrier to expansion; and the high cost of HCV treatment as a barrier to the implementation of treatment recommendations at spoke sites.

Programmes reported a variety of plans and directions for the future, mainly surrounding programme expansion. Most programmes reported plans to increase their number of spoke sites, with several programmes targeting their expansion towards specific clinical sites, such as mobile clinics and those serving indigenous populations. To promote expansion, several programmes plan to apply for additional funding, or for an ECHO Superhub designation, defined as an organization that can train and support other ECHO partner hubs while maintaining adherence to the ECHO model [48]. A large proportion of programmes also reported that they planned to expand into other liver disease or infectious disease areas, including hepatocellular carcinoma, non-alcoholic steatohepatitis, HIV pre-exposure prophylaxis, and sexually transmitted infections.

To improve their current ECHO sessions, several programmes were hoping to increase the quality and variety of their brief didactic lectures, and one programme aimed to create an online resource platform for ECHO participants. Several programmes sought to improve their tracking of recommendations and clinical questions asked, while others aimed to perform qualitative research on their ECHO programme in the future. Two programmes mentioned that they hoped to start offering continuing medical education credits for their spoke site participants, and several programmes were planning to include a broader array of healthcare providers (e.g. pharmacists, nurses, laboratory personnel) in their ECHO sessions.

## Discussion

To our knowledge, this is the first publication to describe key operational and programmatic features of global ECHO programmes for viral hepatitis. Our findings highlight the critical role telementorship can play in increasing capacity for viral hepatitis treatment around the world, and how the Project ECHO model can be successfully implemented across LMICs, as well as high-income countries. Findings from this study are particularly pertinent to the current viral hepatitis elimination landscape, as telementorship has emerged as a successful approach to mitigate the impact of the COVID-19 pandemic on healthcare delivery and represents one of many strategies to help achieve WHO viral hepatitis elimination by 2030 [35, 52–55].

In our study, ECHO hub leaders consistently emphasized the key role telementorship plays in increasing the ability, confidence, and capacity of spoke site providers to treat viral hepatitis. The effectiveness of spoke site providers to treat viral hepatitis has been confirmed in several studies of the Project ECHO model, which showed comparable rates of sustained virologic response following HCV treatment for patients treated at the academic hub versus those treated at ECHO spoke sites [17, 56, 57]. This was further supported by evidence from a large systematic review of 142 studies, which found higher rates of viral load testing, linkage to care and treatment uptake with full decentralisation of care, and comparable cure rates with care delivered by non-specialist physicians and nurses as compared to specialist hepatologists [58–60]. Similar studies have shown that Project ECHO programmes result in enhanced capacity within primary care to treat HCV [30, 61], while data from the U.S. Veterans Health Administration showed that patients with a primary care provider who participates in ECHO are significantly more likely to be initiated on treatment for HCV compared to patients whose primary care provider does not participate in ECHO [62]. Similarly data also exist for HIV, where Project ECHO has been associated with improvement in viral suppression, in both high-income and LMIC settings [33, 63]. It is important to note, however, that the majority of published literature regarding viral hepatitis ECHO programmes are based on HCV experience. To our knowledge, there are no published data on patient level outcomes on the use of telementorship to treat chronic HBV.

In addition to well documented gains in provider knowledge across several different studies of Project ECHO programmes, including those addressing viral hepatitis, there are also benefits in improved provider self-efficacy and job satisfaction [18, 22, 29, 31, 32, 64]. These findings of improvement in provider knowledge and self-efficacy have also been reported with participation in non-viral hepatitis Project ECHO telementorship programmes, including those focusing on HIV, HIV pre-exposure prophylaxis (PrEP), geriatrics, chronic pain management, and neurologic disease [22, 26, 65, 66]. These gains in provider knowledge across a broad spectrum of diseases highlight the common benefits of the learning environment and platform created through the Project ECHO model, and the effectiveness of this model in supporting continuing professional development for healthcare workers [64, 67].

Interestingly, our study found that ECHO leaders identified very similar challenges across geographically and clinically diverse programme sites. Programmes in both high-income countries and LMICs identified securing ongoing funding support as a major challenge and barrier to ongoing success. Programmes that were able to support a part-time administrator consistently highlighted the value of this position, while those programmes that lacked such support identified this as something that would significantly benefit their programme. Several programmes reported receiving one-time grants to support establishment of their ECHO programme, frequently from the pharmaceutical industry. However, these awards were often unable to support ongoing efforts and protected staff time, something that is critical to the growth of any operation. The ongoing issues with funding experienced across a diverse group of programmes speaks to the need to establish ongoing revenue streams for telementorship programmes, including through governmental support and longer-term grant funding from both the public and private sector. Countries such as Georgia and South Africa have taken steps to promote the ECHO model at a national level, by including telementorship within their national plans for viral hepatitis elimination [51, 68]. However, surveyed programmes reported that, even within these countries, waning support for telementorship and a current lack of available government funds remain barriers to programmatic growth.

ECHO hub leaders similarly discussed the challenges associated with a lack of protected time for spoke site participants to attend ECHO clinics. As with funding, this was widely commented upon across a diverse spectrum of programmes, both in high and low-and-middle-income countries and is in keeping with prior literature on barriers to participation in Project ECHO [31, 69–71]. While operationalising protected time for spoke site participants to attend ECHO programmes across a variety of medical reimbursement structures would be challenging, it is important to profile the impact Project ECHO can have on the provision of affordable, quality care, and to consider ways in which participation in ECHO learning initiatives can be supported, both from a clinical time perspective and through reimbursement and payment structures. To support this effort, further data on the clinical and programmatic benefits and cost effectiveness of Project ECHO, particularly in lower-resourced settings, is needed [72].

Despite challenges with funding and protected time for both hub leaders and spoke site providers, it is encouraging that all surveyed programmes reported plans to expand their reach and scope. Expansion of telementorship services is particularly critical in the wake of the COVID-19 pandemic, where social distancing guidelines limited the feasibility of large-scale in-person trainings, and collective experience with remote forms of learning increased. Furthermore, as we move towards the goal of viral hepatitis elimination, there is a tremendous opportunity to leverage and build upon the existing experience with ECHO programmes, expanding these knowledge sharing networks to new geographic locations and different cadres of healthcare workers. Along with expansion of ECHO programmes, there are also opportunities to couple telementorship programmes with additional modalities for healthcare worker training, including online curricula, workshops, and mobile messaging groups, in an effort to support comprehensive and tailored learning. Finally, as DAA therapy for HCV becomes more available and streamlined on a global scale, additional efforts are needed to leverage the success of knowledge sharing networks, such as Project ECHO, to further address the complexities of chronic HBV management, and the management of advanced liver disease.

There are several limitations to our study. First and foremost, this study was performed immediately before the COVID-19 pandemic, which led to massive disruptions in healthcare service delivery as well as considerable expansion of telehealth services and remote learning. While we attempted to identify all viral hepatitis ECHO programmes outside of the U.S. at the time of data collection, we recognize that we likely missed programmes, particularly those who may not have had the funding to attend a formal Project ECHO immersion and are therefore unknown to the Project ECHO team. Furthermore, our findings do not include new programmes that were developed since the start of the COVID-19 pandemic. Additionally, although survey and interview questions were developed by study authors with expertise in the subject area, there were no prior surveys or interview guides of global Project ECHO programmes from which we could model our questions, and surveys and interviews were not piloted or reviewed by external content experts prior to implementation. Additionally, interviews were conducted and analysed by one individual, introducing the potential for subjectivity and bias based on the individual’s prior experiences and beliefs surrounding Project ECHO. Our results are similarly subject to some degree of sampling bias, as only 11 of 18 invited programmes responded to our survey and completed the follow-up interview. This may limit the generalizability of our findings, particularly as programmes with more time and resources may have been more likely to respond. Our small sample size also makes it challenging to effectively compare the telementorship experience across high-income and low-and-middle-income countries, or across different WHO regions. Furthermore, we did not differentiate between HBV and HCV when asking about national plans for viral hepatitis elimination and the provision of viral hepatitis treatment through the public versus private sector, and as such, we may have missed important gaps in funding for HBV specifically, which has historically trailed that of HCV. Future surveys may consider evaluating a variety of telementorship programmes, including those focused on HIV, tuberculosis, and other complex disease processes, to expand the sample size and better understand the ECHO experience across differing disease areas, geographic regions and socioeconomic strata.

## Supplementary Information

**Additional file 1: Appendix A.** Good Practices in Training the Healthcare Workforce: Global survey of viral hepatitis ECHO programmes.

**Additional file 2: Appendix B.** Qualitative Survey.

## Data Availability

Survey results and findings from qualitative interviews are housed within the University of Washington REDCap and are available from the corresponding author upon reasonable request.
