# Supplementary material for: Training the healthcare workforce: the global experience with telementorship for hepatitis B and hepatitis C
Source: BMC Health Serv Res. 2023 Aug 2;23:824. doi: 10.1186/s12913-023-09849-y (PMC10394928; doi:10.1186/s12913-023-09849-y)
Supplement: Supplementary file 1 — Additional file 1: Appendix A. Good Practices in Training the Healthcare Workforce: Global survey of viral hepatitis ECHO programmes. [file 12913_2023_9849_MOESM1_ESM.pdf]

Good Practices in Training the Healthcare Workforce: Global survey of viral hepatitis ECHO programmes

Dear ECHO team,

Thank you for participating in the WHO's survey on good practices in training the healthcare workforce. The goal of this survey is to collect key characteristics, operational features and experiences of different viral hepatitis ECHO programmes around the world.

This survey is being administered in conjunction with a short 30 minute interview, which will take place after the survey is completed.

We appreciate your involvement in this important project and look forward to speaking with you.

CONTACT INFORMATION

Name of person completing survey

____
(e.g. Maria Fernandez)

Role of person completing survey

____
(e.g. ECHO team lead, administrator, etc)

Name of institution

____
(e.g. University of Cape Town)

Name of ECHO programme

____
(e.g. Namibia Ministry of Health HIV ECHO Program)

Name of ECHO programme lead

____
(e.g. Dr. Nandita Mani)

Email fo ECHO programme lead

____
(e.g. mani@uw.edu)

DESCRIPTION OF ECHO CLINICAL SETTING

Please describe the size and scope of the clinical practice at your ECHO hub.	__________________________ (e.g. We are a teaching hospital gastroenterology service with X thousand outpatient visits per year and X inpatients. We have X hepatitis clinics per week that see around X number of HBV and HCV patients. Over the last year X persons received DAA treatment.)
From how many hospital or primary care clinics do you receive referrals? Please provide a number.	__________________________ (e.g. 20)
Approximately how many SPECIALIST PHYSICIANS are in your hepatitis clinical service? Please provide a number.	__________________________ (e.g. 15)
Approximately how many GENERAL PHYSICIANS are in your hepatitis clinical service? Please provide a number.	__________________________ (e.g. 10)
Approximately how many NURSES are in your hepatitis clinical service? Please provide a number.	__________________________ (e.g. 15)

CURRENT STATUS OF NATIONAL HEPATITIS RESPONSE

Please describe the current status of your country's national hepatitis response.

____
(e.g. Is there a plan for viral hepatitis elimination? Are there designated testing and treatment sites? Is there a training programme for health workers?)

Is most of the viral hepatitis treatment in your country happening in the public sector (e.g. government run hospitals and clinics) or in the private sector (e.g. private hospitals and clinics)?

- ◯ Public sector
- ◯ Private sector
- ◯ Both public and private sectors

Who is able to prescribe HBV treatment in your country (select all that apply):

- ☐ Liver specialists
- ☐ Infectious disease specialists
- ☐ General practitioners and/or primary care doctors
- ☐ Nurses
- ☐ Pharmacists
- ☐ Other

Please specify:

____

Who is able to prescribe HCV treatment in your country (select all that apply):

- ☐ Liver specialists
- ☐ Infectious disease specialists
- ☐ General practitioners and/or primary care doctors
- ☐ Nurses
- ☐ Pharmacists
- ☐ Other

Please specify:

____

Which hepatitis C antiviral regimens are in use in your country (select all that apply):

- ☐ Glecaprevir-Pibrentasvir
- ☐ Sofosbuvir-Velpatasvir
- ☐ Ledipasvir-Sofosbuvir
- ☐ Sofosbuvir-Velpatasvir-Voxilaprevir
- ☐ Sofosbuvir and Daclatasvir
- ☐ Elbasvir-Grazoprevir
- ☐ Interferon and Ribavirin
- ☐ Other

Please specify:

____

Which hepatitis B antiviral regimens are in use in your country (select all that apply):

- ☐ Tenofovir disoproxil fumarate
- ☐ Tenofovir alafenamide fumarate
- ☐ Entecavir
- ☐ Other

Please specify:

____

Which of the following diagnostic tests are in use for HBV in your country (select all that apply):	☐ Laboratory-based immunoassay for hepatitis B surface antigen<br>☐ Point of care rapid diagnostic testing for hepatitis B surface antigen<br>☐ Dried blood spot for hepatitis B surface antigen<br>☐ Alanine aminotransferase level<br>☐ Laboratory-based nucleic acid testing for hepatitis B DNA<br>☐ GeneXpert for hepatitis B DNA<br>☐ Dried blood spot for hepatitis B DNA<br>☐ Immunoassay for hepatitis E antigen
Which of the following diagnostic tests are in use for HCV in your country (select all that apply):	☐ Laboratory-based immunoassay for hepatitis C antibody<br>☐ Point of care rapid diagnostic testing for hepatitis C antibody<br>☐ Laboratory-based nucleic acid testing for hepatitis C RNA<br>☐ GeneXpert for hepatitis C RNA<br>☐ Assay for hepatitis C core antigen

DESCRIPTION OF ECHO PROGRAMME AND ACTIVITIES

Where is your ECHO hub located? Please select the best answer.

- ◯ National ministry of health
- ◯ Provincial / state ministry of health or health department
- ◯ Academic medical center
- ◯ Private clinical hospital or practice
- ◯ Non-governmental organization
- ◯ Private-public partnership organization
- ◯ Other

Please specify:

____

In which city is your ECHO Hub located?

____
(e.g. Istanbul)

Is your ECHO program multi-country, national, regional, or local (please select one):

- ◯ Multi-country
- ◯ National
- ◯ Regional
- ◯ Local

In what year did your ECHO program start?

- ◯ 2003
- ◯ 2004
- ◯ 2005
- ◯ 2006
- ◯ 2007
- ◯ 2008
- ◯ 2009
- ◯ 2010
- ◯ 2011
- ◯ 2012
- ◯ 2013
- ◯ 2014
- ◯ 2015
- ◯ 2016
- ◯ 2017
- ◯ 2018
- ◯ 2019

Does your program address HBV, HCV, or both?

- ◯ HBV only
- ◯ HCV only
- ◯ HBV and HCV

How many total spoke sites did you start with?

____
(e.g. 3)

How many total spoke sites do you currently have?

____
(e.g. 20)

What percentage of your spoke sites are urban or semi-urban?

____
(e.g. 75%)

What percentage of your spoke sites are rural?

____
(e.g. 25%)

DESCRIPTION OF ECHO PROGRAMME AND ACTIVITIES, CONTINUED

What type of spoke sites does your ECHO program train? Please select all that apply.	☐ Primary care / general medicine clinics<br>☐ Specialty clinics<br>☐ Harm reduction centers / syringe service programmes<br>☐ Addiction medicine clinics / substance use clinics<br>☐ Public health departments<br>☐ Private hospitals<br>☐ Government hospitals<br>☐ Academic medical centers<br>☐ Non-governmental organization<br>☐ Jail or prison health clinics<br>☐ Other
Please specify:	______________________________
Are there other ECHO programs at your institution?	◯ Yes<br>◯ No
Please indicated what other ECHO programs are at your institution (select all that apply):	☐ HIV ECHO<br>☐ TB ECHO<br>☐ Laboratory ECHO<br>☐ Cancer ECHO<br>☐ Other
Please list other ECHO programs at your institution:	______________________________________

OPERATIONAL ASPECTS

What is the frequency of your teleECHO clinics?
- ◯ Weekly
- ◯ Twice a month
- ◯ Monthly
- ◯ Other

Please specify

____

Please list the videoconferencing platform you use:

____
(e.g. Zoom, GoToMeeting, WebEx)

Please describe the duration and general structure of your teleECHO clinics:

____
(e.g. Clinics are 1 hour long with a short 20-minute didactic session followed by clinical case discussions)

Who leads your teleECHO clinics?
- ◯ Hepatologist
- ◯ Infectious diseases specialist
- ◯ General physician and/or primary care physician
- ◯ Nurse
- ◯ Pharmacist
- ◯ Other

Please specify

____

During teleECHO clinics, who provides advice from the hub site (e.g. who is on the expert panel)? Please select all that apply.
- ☐ Hepatologist
- ☐ Infectious disease physician
- ☐ General physician and/or primary care physician
- ☐ Addiction medicine physician
- ☐ Nurse
- ☐ Pharmacist
- ☐ Social worker
- ☐ Community health worker
- ☐ Other

Approximately how many hepatologists participate? Please provide a number.

____
(e.g. 2)

Approximately how many infectious disease physicians participate? Please provide a number.

____
(e.g. 1)

Approximately how many general physicians and/or primary care physicians participate? Please provide a number.

____
(e.g. 3)

Approximately how many addiction medicine physicians participate? Please provide a number.

____
(e.g. 1)

Approximately how many nurses participate? Please provide a number.	______________________ (e.g. 2)
Approximately how many pharmacists participate? Please provide a number.	______________________ (e.g. 2)
Approximately how many social workers participate? Please provide a number.	______________________ (e.g. 1)
Approximately how many community health workers participate? Please provide a number.	______________________ (e.g. 3)
Please specify the type and number of other health care professionals who participate in the hub expert panel:	______________________
On average, how many spoke site participants attend each teleECHO clinic? Please list a number.	______________________ (e.g. 30)
In general, what type of providers at the spoke site attend each teleECHO clinic (select all that apply):	☐ General practitioners / primary care doctors<br>☐ Liver specialists<br>☐ Infectious diseases specialists<br>☐ Pediatricians<br>☐ Nurses<br>☐ Pharmacists<br>☐ Social workers<br>☐ Community health workers<br>☐ Medical or other health professional students<br>☐ Other
Please specify:	______________________
Approximately how many people total (at the spoke sites) have participated in your ECHO program since it started? Please list a number.	______________________ (e.g. 350)
What are the most common clinical questions that come up during your ECHO sessions. Please describe:	______________________ (e.g. 1. How do I interpret my patient's APRI score? 2. Does my patient have cirrhosis? 3. Should I start my patient on treatment for chronic hepatitis B? 4. Are there any drug-drug interactions between my patient's chronic medications and the available DAA regimens?)
Are you using a supplemental mobile messaging groups, such as WhatsApp, to provide additional consultative support?	◯ Yes<br>◯ No

Please describe how you are using mobile messaging groups:	__________________________________<br>(e.g. All ECHO spoke site participants are added to a WhatsApp group, where they can ask clinical questions to hub experts and each other between sessions.)
As part of your teleECHO program, do spoke sites receive any additional viral hepatitis education?	◯ Yes, we also provide educational workshops<br>◯ Yes, we also provide online trainings<br>◯ Yes, we provide both workshops and online trainings<br>◯ Yes, we provide another form of training.<br>◯ No
Please specify:	__________________________________
Do your ECHO participants receive continuing education credits or a certificate for participating in the ECHO program? Please select all that apply.	☐ Yes, a certificate of competency is awarded<br>☐ Yes, a certification for antiviral prescribing from the ministry of health or another accrediting body is awarded.<br>☐ Yes, CME is provided<br>☐ No
Did anyone from your program attend the 3-day ECHO Immersion at the University of New Mexico or at another ECHO Superhub?	◯ Yes<br>◯ No<br>◯ Unknown
Have you received any funding to support your ECHO programme?	◯ Yes<br>◯ No
Please specify funding source:	__________________________________
Have you kept any data on your program?	◯ Yes<br>◯ No
What type of data to you have on your ECHO program?	__________________________________<br>(e.g. SVR rates of spoke site patients, number of patients served, most common questions asked)

REFLECTIONS

Please list what you see as the main positive features of your ECHO program. What has worked particularly well for your program? ____

Please list what has not worked well for you program. What have been the major challenges you have encountered in running your ECHO program, and how you have you sought to overcome these challenges? ____

What plans do you have for the future to expand and/or improve your program? ____
