# Supplementary material for: Training the healthcare workforce: the global experience with telementorship for hepatitis B and hepatitis C
Source: BMC Health Serv Res. 2023 Aug 2;23:824. doi: 10.1186/s12913-023-09849-y (PMC10394928; doi:10.1186/s12913-023-09849-y)
Supplement: Supplementary file 2 — Additional file 2: Appendix B. Qualitative Survey. [file 12913_2023_9849_MOESM2_ESM.pdf]

Qualitative Survey

Record ID

____

Name of ECHO Interviewee

____
(e.g. Maria Corcorran)

Name of ECHO Program

____
(e.g. UW Hep C ECHO)

Why did you establish an ECHO program?

____

How does your ECHO programme fit into a national or regional programme for viral hepatitis elimination?

____

How has your programme evolved since its inception?

____

Do you train all cadres of healthcare workers/ providers in one ECHO session, or do you have separate sessions?

____

Approximately how many hours per week do you and your staff spend on your Project ECHO programme?

____

Do you keep information on the clinical questions and scenarios asked? If so, how is this information used?

____

What other types of data do you have on your programme?

____

What were some of the major challenges in setting up and implementing the programme, and how have you overcome them?

____

What have been some of the major challenges and barriers to success?

____

What do you see as the main positive features of your ECHO programme:

____

What do you see as the future directions of your ECHO programme?

____

Other comments:

____
